# Beta-Catenin and Plakoglobin Expression during Zebrafish Tooth Development and Replacement

**DOI:** 10.1371/journal.pone.0148114

**Published:** 2016-03-03

**Authors:** Barbara Verstraeten, Jolanda van Hengel, Ann Huysseune

**Affiliations:** 1 Evolutionary Developmental Biology, Ghent University, Ghent, Belgium; 2 Molecular Cell Biology Unit, Department for Molecular Biomedical Research, VIB Ghent, Ghent, Belgium; 3 Department of Biomedical Molecular Biology, Ghent University, Ghent, Belgium; Deakin School of Medicine, AUSTRALIA

## Abstract

We analyzed the protein distribution of two cadherin-associated molecules, plakoglobin and β-catenin, during the different stages of tooth development and tooth replacement in zebrafish. Plakoglobin was detected at the plasma membrane already at the onset of tooth development in the epithelial cells of the tooth. This pattern remained unaltered during further tooth development. The mesenchymal cells only showed plakoglobin from cytodifferentiation onwards. Plakoglobin 1a morpholino-injected embryos showed normal tooth development with proper initiation and differentiation. Although plakoglobin is clearly present during normal odontogenesis, the loss of plakoglobin 1a does not influence tooth development. β-catenin was found at the cell borders of all cells of the successional lamina but also in the nuclei of surrounding mesenchymal cells. Only membranous, not nuclear, β-catenin, was found during morphogenesis stage. However, during cytodifferentiation stage, both nuclear and membrane-bound β-catenin was detected in the layers of the enamel organ as well as in the differentiating odontoblasts. Nuclear β-catenin is an indication of an activated Wnt pathway, therefore suggesting a possible role for Wnt signalling during zebrafish tooth development and replacement.

## Introduction

During the various phases of development of the vertebrate body, it is of major importance that cells are capable of communicating with each other and of rearranging themselves. Adhesion molecules play a very important role during the morphogenetic processes that underlie development [1–4].

There are different types of cell junctions. Adherens junctions (AJs) are an important type of intercellular junction, consisting of the cadherin-catenin complex. Cadherins represent an important family of cell adhesion molecules, and include classical cadherins and desmosomal cadherins. Classical cadherins are connected to the actin cytoskeleton through β-catenin. At the cell membrane the cadherins cluster laterally to achieve strong adhesive forces. By connecting to cadherins at the neighbouring cell membrane, cells are held closely together [5–7]. Desmosomes represent another type of intercellular junctions. They provide strong intercellular adhesion by linking to the intermediate filament cytoskeleton, and are abundant in tissues subjected to mechanical stress. Transmembrane desmosomal cadherins, i.e., desmocollins and desmogleins, are coupled at the C-terminal end to the linker proteins plakophilin and plakoglobin which together form the desmosomal plaque. This plaque is linked to the intermediate filaments via desmoplakin, forming a characteristic highly organized, electron-dense structure [8, 9].

The cadherin-associated molecules plakoglobin and β-catenin are close homologues. Their sequence is partially similar and they compete for the same binding site at the C-terminal region of classical cadherins [10]. While plakoglobin has the capacity to substitute for β-catenin in AJs, β-catenin can also interact with desmosomal cadherins and therefore replace plakoglobin at the desmosomes but only when excessive desmosomal cadherins are present or when there is no plakoglobin available. These interactions suggest a cross-talk between adherens junctions and desmosomes [11, 12].

Apart from functioning in cell adhesion, β-catenin is also known to play a role in the Wnt pathway by binding to the LEF/TCF binding site in the nucleus [13–15]. Given the similarity between β-catenin and plakoglobin, it is not surprising that plakoglobin can also bind LEF/TCF. However, the role for these two close homologues in the Wnt signalling appears to be distinct. The precise role of plakoglobin in the Wnt signalling pathway is still controversial. The complete knockout (KO) of either β-catenin or plakoglobin in mice, results in a lethal phenotype. Intriguingly, both phenotypes are distinctive of one another. The loss of β-catenin results in animals missing dorsal structures while the plakoglobin null mouse fails to form correct desmosomal structures causing a failure in heart development [16, 17].

Tooth development has been used for many years as a paradigm for investigating organ development. Not only do teeth result from multiple, reciprocal interactions between two tissue layers, the epithelium and underlying mesenchyme, but these two tissue layers undergo extensive morphogenetic movements. Tooth formation starts with the establishment of an epithelial thickening, called placode. As the epithelium and underlying mesenchyme pass through different stages of morphogenesis, both their cells need to rearrange before they can differentiate and deposit tooth-specific matrices [18–20]. How such cell rearrangements are achieved, and what the role of cell adhesion molecules is in this process, has only partially been addressed.

We have set out for a study aiming at elucidating the role of cell adhesion molecules during development and replacement of teeth. To this end, we focus on the zebrafish since this species, contrary to mammalian species, replaces its teeth throughout life. Zebrafish lack jaw teeth in the oral cavity, but have pharyngeal teeth located on the fifth branchial arch. The full zebrafish dentition consists of 11 teeth on each side, divided into three rows, called ventral (V), mediodorsal (MD) and dorsal (D) rows. The first tooth starts to develop at two days post-fertilization (dpf) in position 4 of the ventral row (4V^1^, superscript indicating the generation number). soon followed by the adjacent teeth in the same row (i.e., 3V^1^ and 5V^1^). At around 80 hours post-fertilization (hpf) the first developing tooth attaches to the branchial arch, thereby becoming functional. At the same time, a replacement tooth (i.e., 4V^2^) starts to develop from the base of the crypt surrounding the functional tooth [21].

An earlier study on E-cadherin expression and distribution in zebrafish teeth has shown that the epithelial-derived part of the tooth remains E-cadherin positive throughout development. In contrast, the mesenchymal cells never display any E-cadherin expression [22]. Because of the absence of down-regulation of E-cadherin during the formation of a new tooth, we expand our research to the cadherin-related molecules β-catenin and plakoglobin. In the mammary gland, the formation of an epithelial bud is accompanied with the down-regulation of the desmosomal compartment [23]. On the other hand, studies on cancer and tumor development suggest that the loss of β-catenin might make the cadherin-catenin complex incompetent. This would cause loss of adhesive strength and decrease the number of AJs present. Both β-catenin and plakoglobin are expressed during murine tooth development, but the observations are scattered [24, 25]. In zebrafish, β-catenin and plakoglobin have two paralogues, a result of the genome duplication that has occurred in teleosts. Plakoglobin-1b is a shorter transcript than plakoglobin-1a and up till now it has not been linked to a certain function in zebrafish development. Plakoglobin-1a is described to play a critical role during cardiac development and to have a signalling role during zebrafish development [26]. The two β-catenin genes that have been discovered in zebrafish, β-catenin-1 and β-catenin-2, have distinct roles during zebrafish development [27].

Here we describe the distribution of both β-catenin and plakoglobin during tooth development and tooth replacement, in first- and later-generation teeth. Using data from knockdown experiments, we analyse the possible function of plakoglobin during tooth development.

## Results

### Distribution of plakoglobin during zebrafish tooth development and replacement

**Fig 1:**
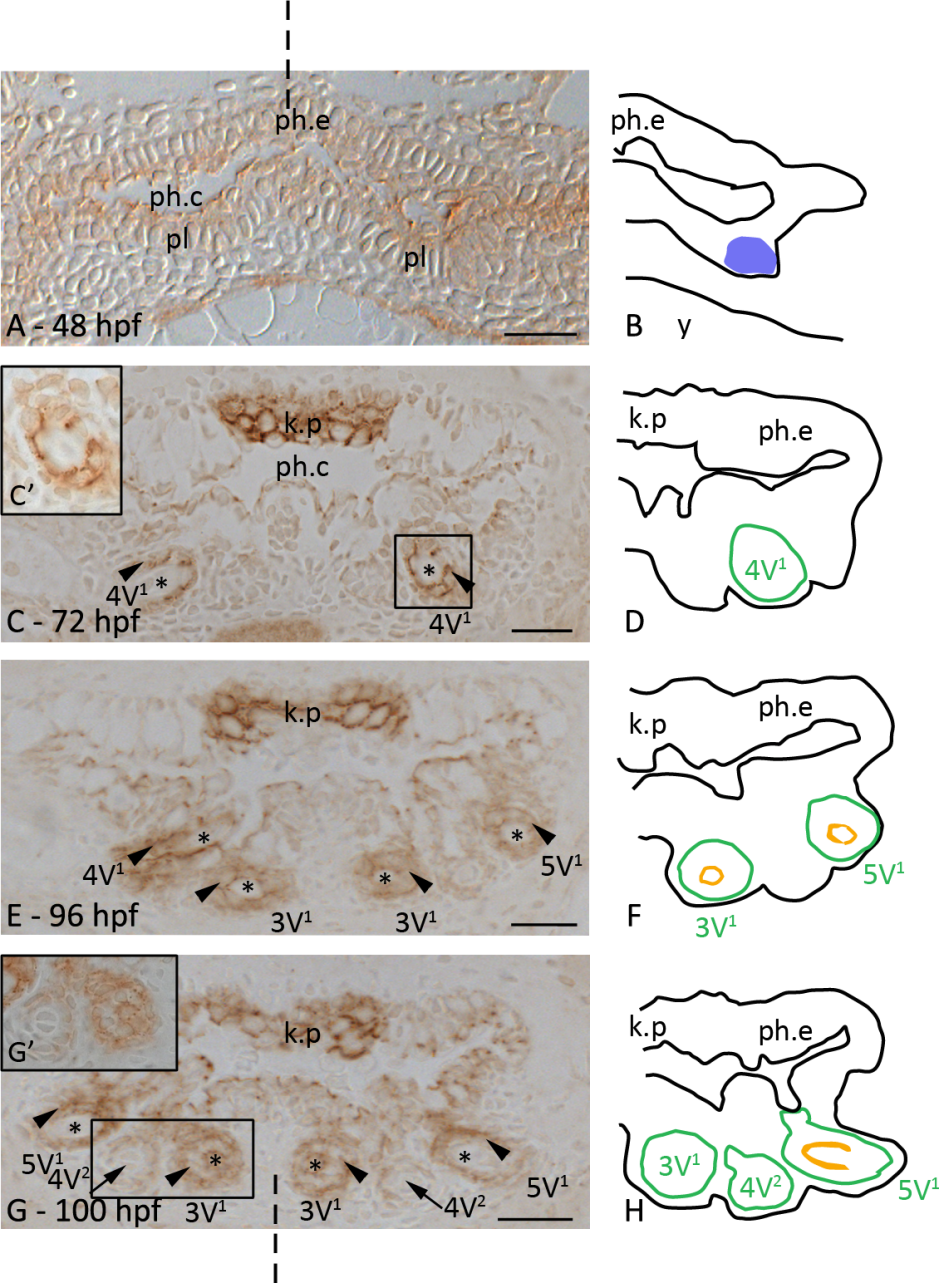
Plakoglobin distribution during the development of first-generation teeth. Cross-sections through the pharyngeal region of a 48 hpf (A), 72 hpf (C), 96 hpf (E) and 100 hpf (G) zebrafish embryo and corresponding schematic drawings in B, D, F and H. A,B: Initiation stage of tooth 4V^1^; the pharyngeal epithelium (ph.e.) expresses plakoglobin, in contrast to the mesenchymal cells. C,D: Morphogenesis stage of tooth 4V^1^; plakoglobin is clearly expressed at the cell borders of the epithelial-derived tissue (arrowhead). The keratinized pad (k.p.) opposite the developing teeth, strongly expresses plakoglobin. Boxed area in C is magnified in C’. E,F: Tooth 4V^1^ in late cytodifferentiation stage; teeth 3V^1^ and 5V^1^ in early cytodifferentiation stage. All teeth present display plakoglobin expression in epithelial-derived cell layers. G,H: Initiation of the first replacement tooth (arrow), 4V^2^. The epithelial outgrowth shows plakoglobin expression while the condensed mesenchyme is negative. Boxed area in G is magnified in G’. Diagrams: blue patch: placode; green line: contour of the tooth; orange: tooth matrix. Orientation: dorsal to the top, ventral to the bottom of each figure; dashed line indicates mediosagittal plane. Additional abbreviations: ph.c: pharyngeal cavity; pl: placode; *: dental papilla. Scale bars = 20 μm.

At 48 hpf, plakoglobin is present in the pharyngeal epithelium as dots on the plasma membrane, restricted to the side facing the pharyngeal lumen. This dotted distribution of plakoglobin typically reflects the distribution of desmosomes. The mesenchymal cells surrounding the pharyngeal epithelium do not show any expression of plakoglobin (Fig 1A and 1B). On both sides of the midline the epithelium lining the floor of the presumptive pharyngeal cavity thickens to form a placode. During morphogenesis, the cells of this placode protrude into the underlying mesenchyme creating the bell-shaped enamel organ of the developing tooth (4V^1^, Fig 1C and 1D, 72 hpf). During this stage, plakoglobin is strongly expressed at the plasmamembrane of the cells of the enamel organ. In contrast, the condensed mesenchyme does not show any plakoglobin expression. In addition, the keratinized pad, forming in the roof of the pharyngeal cavity opposite the site where teeth develop, strongly expresses plakoglobin at the cell membrane. The cells of the pharyngeal epithelium maintain their dotted plakoglobin expression (Fig 1C and 1D).

In 96 hpf specimens, tooth 4V^1^ is flanked by tooth 3V^1^ on its medial and tooth 5V^1^ on its lateral side. Tooth 4V^1^ is now in cytodifferentiation stage, featuring the differentiation of the inner dental epithelium of the enamel organ into ameloblasts and the differentiation of cells of the dental papilla into odontoblasts. Teeth 3V^1^ and 5V^1^ have nearly completed morphogenesis. In all teeth present, the expression pattern of plakoglobin is similar: plakoglobin is strongly expressed in both the inner and the outer dental epithelium while the cells of the mesenchymal dental papilla show a weak expression. The keratinized pad maintains its plakoglobin expression during further development and growth of the embryo (Fig 1E and 1F).

The first replacement tooth, tooth 4V^2^, starts to develop at around 80 hpf. Its initiation coincides with the attachment and eruption of its predecessor. The enamel organ of the replacement tooth expresses plakoglobin (Fig 1G and 1H). The reduced enamel organ of the functional tooth is still expressing plakoglobin but less intense. Teeth 3V^1^ and 5V^1^, now in late cytodifferentiation stage, express plakoglobin in the inner and outer dental epithelium and the differentiating odontoblasts (Fig 1G and 1G’).

**Fig 2:**
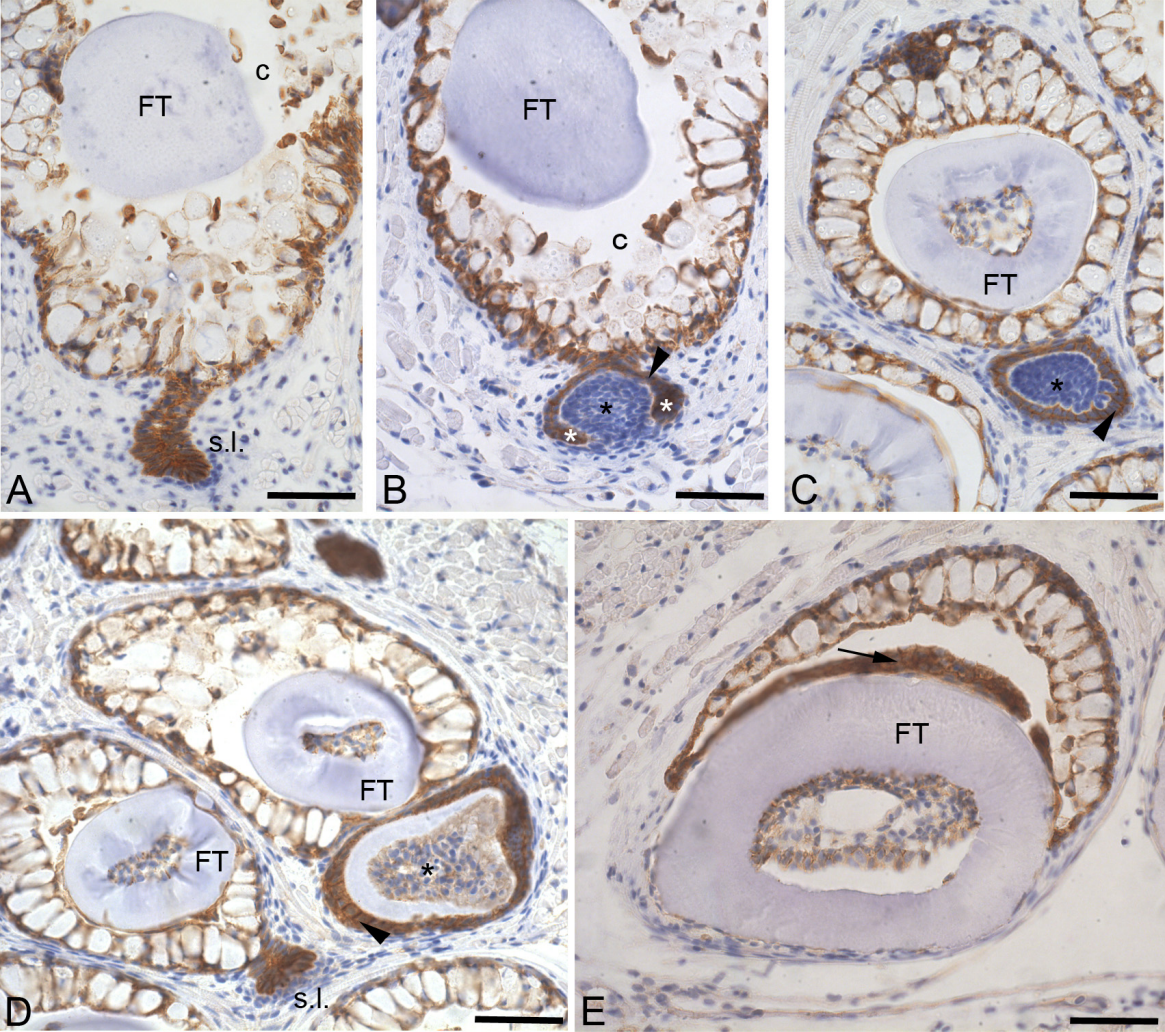
Plakoglobin distribution in adult replacement teeth. Cross-sections through one of the paired fifth branchial arches of an adult zebrafish. A: In contrast to the surrounding mesenchyme, all the cells constituting the successional lamina (s.l.) express plakoglobin at their cell membrane. Also the cells of the crypt epithelium express plakoglobin. B: Morphogenesis stage; the condensed mesenchyme constituting the dental papilla (black asterisk) is plakoglobin-negative. The enamel organ (arrowhead), including cervical loops (white asterisk), is strongly expressing plakoglobin. C: During early cytodifferentiation, the expression of plakoglobin remains limited to the inner and outer dental epithelium (arrowhead). D: Tooth in late cytodifferentiation stage, showing plakoglobin expression in the enamel organ (arrowhead) and in the differentiating odontoblasts lining the dental papilla (asterisk). E: Erupted functional tooth (FT) showing that even in a fully developed tooth plakoglobin expression persists in the reduced enamel organ (arrow). Orientation: dorsal to the top, ventral to the bottom, medial to the right and lateral to the left of the figure. Additional abbreviations: c: crypt surrounding the tip of the functional predecessor. Scale bars = 50 μm.

Unlike the formation of a first-generation tooth, the initiation of a replacement tooth starts with the formation of a so-called successional lamina, an outgrowth of the crypt epithelium surrounding the functional tooth. A successional lamina cannot yet be distinguished in the first replacement tooth 4V^2^, but becomes prominent in larger juveniles and adults. We have examined expression of plakoglobin in adult jaws, where the teeth have cycled through many generations. The cells of the successional lamina express plakoglobin very strongly at the cell membrane while the mesenchymal tissue does not (Fig 2A). As tooth development progresses, plakoglobin expression remains restricted to the epithelial-derived part of the tooth. During morphogenesis stage, all cells of the epithelial enamel organ express plakoglobin. There is no difference of intensity between the cervical loop and the rest of the enamel organ. The now clearly condensed mesenchymal cells do not express plakoglobin (Fig 2B). When cytodifferentiation starts, there is no visible change in expression of plakoglobin in the enamel organ. Both inner and outer dental epithelium express plakoglobin equally strongly at the plasma membrane. The differentiating odontoblasts also express plakoglobin, as do the other cells of the dental papilla (Fig 2C and 2D). After attachment and eruption, the reduced enamel organ still expresses plakoglobin (Fig 2E). Throughout the different stages of tooth development, the cells of the epithelial crypt surrounding the functional tooth consistently express plakoglobin, with no visible variation in intensity or localisation of the signal. The mesenchymal tissue surrounding the crypts and the developing teeth never expresses plakoglobin during any stage of tooth development. In summary, both in first-generation and later-generation teeth plakoglobin is present in the different layers of the developing enamel organ. The mesenchymal-derived cells only show a weak signal for plakoglobin from cytodifferentiation onwards. The cells of the successional lamina strongly express plakoglobin, suggesting that the formation of a new tooth is not accompanied by the loss of this desmosomal component.

### Tooth development in plakoglobin morphant zebrafish

Given the sustained presence of plakoglobin in the epithelial enamel organ, and its presence in the mesenchyme from cytodifferentiation onwards, we wished to determine the role of plakoglobin during zebrafish tooth development and/or replacement.

Plakoglobin-morpholino injected zebrafish have been reported to display a delayed midbrain-hindbrain border formation, reduced heart size and kinked tail [26, 28]. No attention was paid so far to the tooth phenotype in these knockdown experiments.

**Fig 3:**
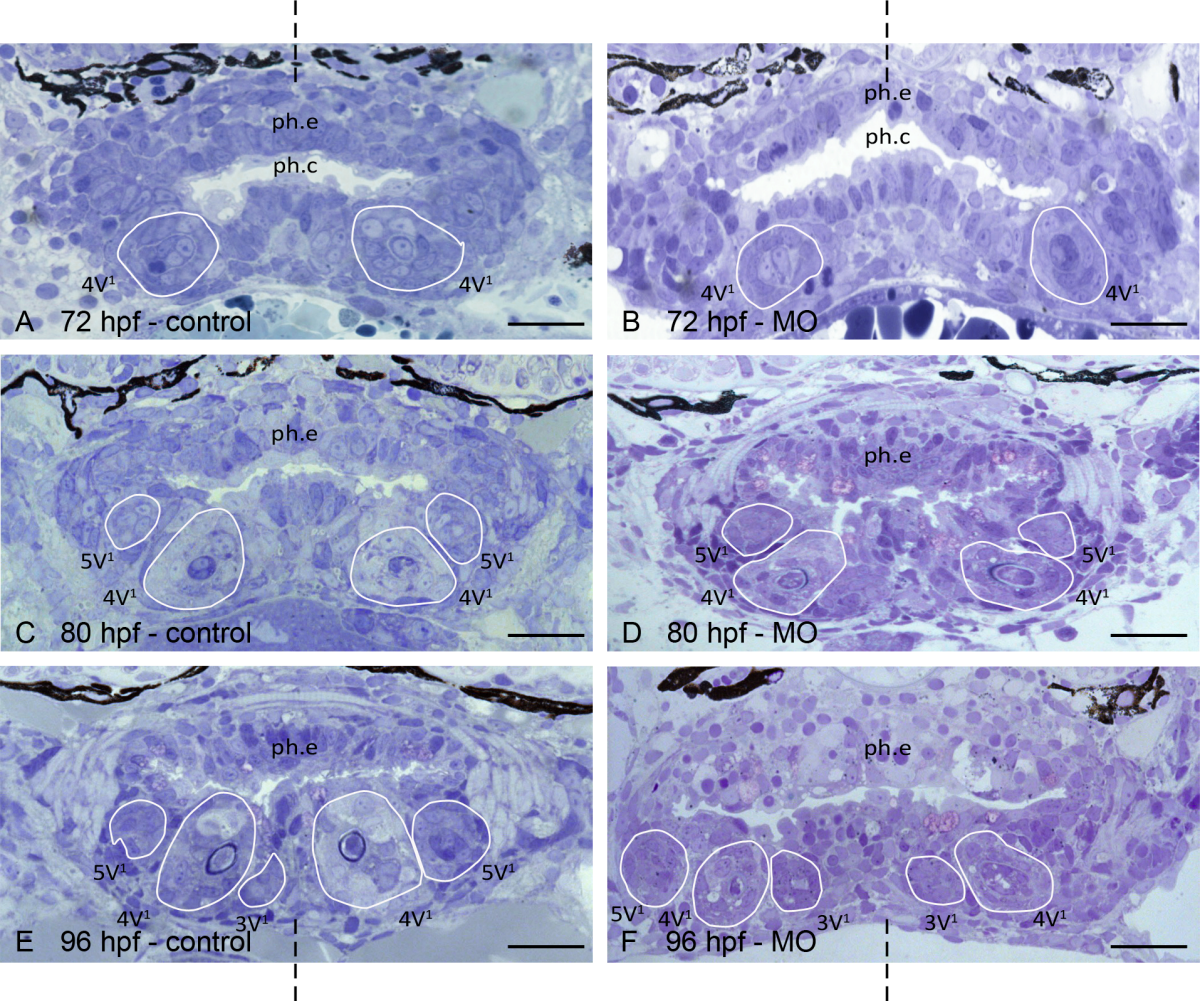
Comparing tooth development in control-injected and plakoglobin morpholino-injected zebrafish. A,C,E: cross-sections through the pharyngeal cavity (ph.c.) of control-injected zebrafish at 72, 80 and 96 hpf, respectively; B,D,F: cross-sections at approximately the same level in plakoglobin morphant (MO) zebrafish at 72, 80 and 96 hpf, respectively. A,B: In both control-injected and morphant embryos the first tooth to develop, 4V^1^ is in early cytodifferentiation stage. C,D: Tooth 4V^1^ has continued to develop in control-injected and morphant embryos. Moreover, tooth 5V^1^ is also visible in both dentitions. E,F: The first three tooth positions develop normally in both control-injected and in morphant zebrafish. Orientation: dorsal to the top, ventral to the bottom of each figure; dashed line indicates mediosagittal plane. Additional abbreviations: ph.e: pharyneal epithelium, line: contour of the developing tooth. Scale bars = 20 μm.

Control-injected as well as morphant embryos are both delayed in development compared to wild-type embryos, as indicated by retardation in cranial cartilage differentiation, yolk resorption and pharyngeal lumen formation. At 72 hpf, control-injected and morphant embryos display the first tooth, 4V^1^, on both sides of the midline in early cytodifferentiation stage. In morphants, tooth 4V^1^ develops at the right position, and shows differentiation of the enamel organ into inner and outer dental epithelium. Also the start of formation of matrix is observed in the morphant embryos (Fig 3A and 3B).

Morphant embryos at 80 hpf display tooth 4V^1^ and 5V^1^, as in control embryos, with tooth 4V^1^ in late and tooth 5V^1^ in early cytodifferentiation stage (Fig 3C and 3D). At 96 hpf, all three first tooth loci are occupied and the teeth do not display any abnormalities (Fig 3E and 3F). Thus, in the absence of a functional plakoglobin protein, the teeth of the first generation still develop, their enamel organ differentiates and the ameloblasts can produce a normal amount of tooth matrix (enameloid) in conjunction with the odontoblasts. Given the general developmental delay both in control and morphant embryos compared to wild-type, and given that embryos did not survive beyond 96 hpf, we were unable to assess the effect of plakoglobin knockdown in replacement tooth formation.

### Distribution of β-catenin during zebrafish tooth development and replacement

The data concerning the distribution of β-catenin during tooth development are based on immunostaining of paraffin sections of adult jaws and are therefore limited to replacement teeth.

**Fig 4:**
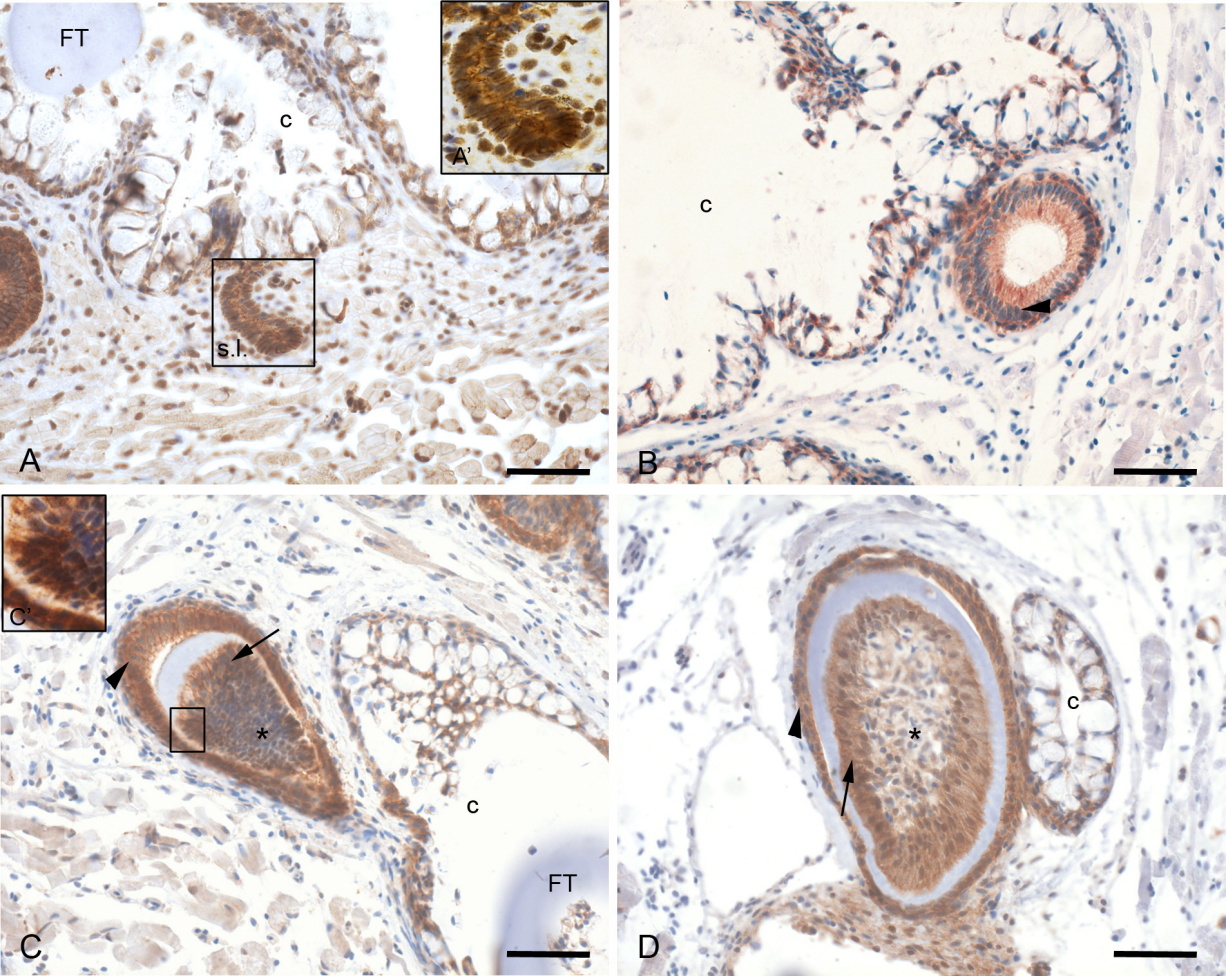
β-catenin distribution during the development of adult replacement teeth. A,A’: The successional lamina (s.l.) shows expression of β-catenin at the cell membrane and in some nuclei. The mesenchyme also displays nuclear β-catenin. B: β-catenin is detected only at the plasma membrane of the epithelial cells during morphogenesis stage (arrowhead). C: In the enamel organ (arrowhead) β-catenin is expressed at the plasma membrane of cells of both inner and outer dental epithelium. C’: The dental papilla (asterisk) shows odontoblasts with membrane-bound and nuclear β-catenin expression. D: During late cytodifferentiation stage, β-catenin remains expressed in both the inner and outer dental epithelium as well as in the polarized odontoblasts (arrow) adjoining the tooth matrix. Orientation: dorsal to the top, ventral to the bottom, medial to the right and lateral to the left of the figure. Additional abbreviations: c: crypt slightly posterior to the tip of the functional predecessor; FT: functional tooth. Scale bars = 50 µm.

β-catenin is expressed at the cell membrane of all cells constituting the successional lamina, but not of the mesenchymal cells. However, both in the epithelial as well as in the mesenchymal cells of the developing replacement tooth, β-catenin is present in some nuclei (Fig 4A and 4A’).

During morphogenesis stage, when the epithelium further invaginates and the mesenchyme condenses, β-catenin is expressed in the enamel organ but not in the condensed mesenchyme. Both the inner and outer dental epithelium express β-catenin at the plasma membrane. β-catenin is however no longer observed in the nuclei (Fig 4B).

Once cytodifferentiation starts, the expression of β-catenin expands. While β-catenin remains expressed at the plasma membrane in the inner and outer dental epithelium, it is now also expressed in the differentiated, polarized odontoblasts that line the dentine matrix. In these cells β-catenin is expressed both at the cell membrane and in the nucleus. The β-catenin signal in the centre of the dental papilla is either very low or completely lacking (Fig 4C and 4C’).

In the functional tooth, β-catenin is expressed at the plasma membrane of the cells of the reduced enamel organ as well as in the odontoblasts, both in the nucleus and at the plasma membrane. The expression is much weaker in the centre of the dental papilla (now called dental pulp). β-catenin is also expressed at the plasma membrane of the cells constituting the epithelial crypts surrounding the functional tooth (Fig 4D).

## Discussion

In this study we show the distribution of two closely related cell adhesion molecules during the development and replacement of zebrafish teeth. β-catenin and plakoglobin are expressed by the same cell layers, but β-catenin has a broader distribution. Plakoglobin expression is restricted to the epithelial-derived part of the tooth during initiation and morphogenesis stage but expands to the dental papilla from cytodifferentiation onwards. β-catenin is also expressed both in the epithelial enamel organ and in the mesenchymal-derived dental papilla. However, unlike plakoglobin, β-catenin is also found in the nuclei of cells of the enamel organ and of differentiating odontoblasts. The analysis of plakoglobin morphant embryos revealed no abnormalities as the first three teeth developed normally.

The scarce data available on plakoglobin distribution in teeth are limited to the mouse model, where its expression parallels that observed here in zebrafish. In both species, plakoglobin is expressed in the epithelial part of the developing tooth. In mice, an asymmetrical distribution has been observed, the plakoglobin protein signal being weaker on the medial side of the tooth [24, 29]. While the epithelial downgrowth that produces the tooth in zebrafish is skewed towards the future attachment site [30], and some proteins are unevenly distributed on both sides of the tooth during cytodifferentiation [22] an uneven distribution of plakoglobin is not observed.

The distribution of β-catenin during tooth development has been studied previously in mice and in humans [25, 31–33]. Both in mice and in humans, the enamel organ is expressing β-catenin throughout tooth development with a stronger expression of β-catenin in the enamel knot. The enamel knot is known to be a signalling centre which is especially important for cusp formation. In zebrafish teeth, which display only one single cusp, an enamel knot has not yet been identified. Different from the mouse, expression of β-catenin in zebrafish was found in the cells of the dental papilla. In the mouse, the dental papilla is expressing β-catenin weakly and the differentiating odontoblasts have been reported to be negative. In human teeth, however, β-catenin expression is observed in the dental papilla and the odontoblasts [25, 31, 32], resembling the situation in zebrafish.

In previous studies, we have shown that both first-generation or later-generation zebrafish teeth form without a detectable down-regulation of E-cadherin [22, 34], refuting a hypothesis proposed by Jamora et al. (2003), accounting for the morphogenesis of an epithelial bud. In the absence of a detectable down-regulation of E-cadherin, how does the placode progress into a downgrowth eventually forming a bell? Two possible explanations have been suggested with regard to different epithelial appendages. In early morphogenesis of the mammary gland and of hair placodes in mice, E-cadherin and β-catenin are present. Yet, instead of a decrease in number of adherens junctions (AJs), the desmosomal components (such as desmoglein, desmocollin, plakoglobin and desmoplakin) are down-regulated during early development [23, 35]. In zebrafish, plakoglobin expression persists in the enamel organ throughout tooth development, suggesting that desmosomes are not disassembled. Second, studies on cancer and tumor development have suggested that the loss of β-catenin could make the cadherin-catenin complex incompetent, causing the loss of adhesive strength [36, 37]. In zebrafish E-cadherin is present throughout tooth development at the plasma membrane, as is β-catenin, suggesting their linkage. Thus, neither a reduction nor a dysfunction of AJs or desmosomes appears to be involved.

Since plakoglobin showed such a strong and specific expression during zebrafish tooth development, plakoglobin morpholino-injected zebrafish were studied. Until 96 hpf, there was no detectable difference between control-injected and morphant larvae. Both morphant and control zebrafish developed teeth at the correct positions. Hence, reduction of plakoglobin did not alter the possibility to initiate and further develop teeth. Furthermore, the morphant teeth were also capable of differentiating, as enameloid was formed. This indicates that plakoglobin is not necessary for the differentiation of the inner dental epithelium or the odontoblasts (which participate in enameloid formation, cf. [38, 39]). Tooth development is not altered despite the loss of an important component of the desmosomes, suggesting that desmosomes do not play a significant role during tooth development or that the absence of plakoglobin 1a is rescued, either by plakoglobin 1b or by β-catenin. Plakoglobin resembles β-catenin as they both contain multiple Armadillo (Arm) repeats. Therefore, β-catenin and plakoglobin functions are possibly redundant. The affinity of β-catenin for binding E-cadherin is much stronger than that of plakoglobin for E-cadherin [40]. Plakoglobin on the other hand has a higher affinity for desmosomal cadherins than for classical cadherins [41]. However, both in cell culture experiments and in KO mice where β-catenin is depleted, plakoglobin compensates for β-catenin in AJs [40, 42]. In the complete absence of plakoglobin, β-catenin can replace plakoglobin in the desmosomes in mice [11]. Given that β-catenin is found in all cells that express plakoglobin, it is possible that β-catenin rescues the integrity of the desmosomes in developing zebrafish teeth by substituting for plakoglobin.

Plakoglobin KO mice die because of severe defects in the developing heart due to the lack of desmosomes. The intestinal epithelium and hair follicle development of these plakoglobin null embryos was not altered and AJs were unaffected demonstrating that epithelial-derived tissues can develop normally without plakoglobin [11, 41]. Cell culture experiments have shown that, when plakoglobin expression was inhibited, the cadherin typical for the cell type remained largely unaffected. Yet, the cells showed a significant reduction of adhesive strength [43]. Thus, without plakoglobin, adhesion can be reduced while at the same time the cadherin-catenin complex remains functional. This could explain why tooth initiation and morphogenesis, which is assumed to require a decrease in adhesion, can progress normally in plakoglobin morphants.

Next to its important function in cell adhesion, β-catenin is also known to be a key mediator of Wnt signalling. The presence of nuclear β-catenin is considered a valid proxy for active Wnt signalling [44]. Several studies have shown the importance of Wnt signalling during tooth development, not only in mice but also in non-mammalians. An active Wnt pathway is necessary for normal murine tooth development [45–52]. A recent study on zebrafish teeth suggests that Wnt signalling is repressed during morphogenesis and cytodifferentiation stage [53]. Our immunohistochemistry results show absence of nuclear β-catenin at morphogenesis stage but presence of nuclear β-catenin from cytodifferentiation onwards in some of the odontoblasts. These observations largely correspond to the data of Huysseune and colleagues (2014) based on dkk1 expression and therefore suggests that also in zebrafish tooth development Wnt signalling is required for normal tooth development [53]. We were unable to determine the role of β-catenin during tooth development using β-catenin MO-injected zebrafish since these do not survive until the start of tooth formation. Likewise, the β-catenin mutant zebrafish *ichabod* is not useful either as their embryos are deficient for maternal, but not zygotically expressed β*-catenin-2* [27].

While β-catenin and plakoglobin share at least some functions, plakoglobin was long thought not to be involved in Wnt signalling. Only later it was discovered that plakoglobin can act on its own in the Wnt signalling pathway by binding to LEF/TCF in the nucleus [8, 54, 55]. As we did not detect plakoglobin in the nucleus, we conclude that tooth development in zebrafish is not influenced by plakoglobin-induced Wnt signalling.

Summarizing, we were able to show the distribution of β-catenin and plakoglobin during the different stages of zebrafish tooth development. These cell adhesion-related molecules were expressed in both the epithelial as well as the mesenchymal component of the developing tooth. Unlike plakoglobin, β-catenin signal could be detected in numerous nuclei suggesting that Wnt signalling is active during initiation but repressed during morphogenesis stage. Although plakoglobin is strongly expressed in developing teeth, the loss of plakoglobin does not seem to affect tooth development as plakoglobin morphants do not show any tooth defect. Neither β-catenin nor plakoglobin is down-regulated during initiation of a new tooth placode. How and which cell adhesion molecules are regulated to obtain the fine balance between reduced adhesion and proper development, needs to be further examined.

## Material and Methods

### Zebrafish collection

Wild-type (wt) zebrafish were raised in a 14h light / 10h dark light regime at the standard temperature of 28.5°C [56]. Embryos, larvae and adults were sacrificed by an overdose of MS222 (3-aminobenzoic acid ethyl ester) according to the Belgian law on the protection of laboratory animals (KB d.d. 13 September 2004). Embryos and larvae were collected every 4 hours, starting at 40 hpf. In addition, fifth branchial arches were dissected from adult zebrafish, using a Leica MZ Apo dissecting microscope and microscissors.

Since euthanasia is not considered an animal experiment, and since developing zebrafish should be regarded as protected animals under the new legislation of animal welfare (EU 2010/63/EU) only from 120 hpf onwards [57], an authorisation of an ethics committee is not available.

### Plakoglobin morphant zebrafish

Plakoglobin morpholino-injected, control morpholino-injected and wild-type zebrafish were obtained from the lab of Dr. M. Grealy (Galway, Ireland). One- to 2-cell stage embryos have been injected with 5 ng of a morpholino constructed to prevent translation of plakoglobin-1a mRNA or with a 5 base pair (bp) mismatch morpholino as control [26]. The batch was raised under standard conditions and sacrificed at specific time points. The knockdown of plakoglobin in morpholino injected embryos was confirmed by western blotting [26].

### Histological analysis of plakoglobin morphants

Plakoglobin MO-injected embryos and larvae were fixed overnight at 4°C in 4% paraformaldehyde (PFA) in phosphate buffered saline (PBS) and embedded in epon according to standard procedures. Semithin (1 μm) sections were made using a diamond knife. Next, the epon sections were stained with toluidine blue (0.5% toluidine blue, 1% borax in bidistilled water) for detailed histological analysis.

### Immunohistochemistry

Wild-type embryos and larvae were fixed overnight at 4°C in 4% PFA in PBS, depigmented, dehydrated in an increasing methanol (MeOH) series and stored in 100% MeOH at -20°C for whole-mount analysis.

The fifth branchial arches dissected from adult zebrafish were fixed overnight at 4°C in 4% PFA in PBS, decalcified in Morse’s solution (10% sodium citrate, 22,5% formic acid) for several days at 4°C, embedded in paraffin according to standard procedures and sectioned at 5 μm with a Prosan Microm HM360 microtome.

To generate data on first-generation teeth and the first replacement tooth (tooth 4V^2^), embryos and larvae were processed for whole mount immunohistochemistry as described previously [58]. Primary antibody used was anti-plakoglobin clone 15 (1/500, BD Transduction Laboratories). This anti-plakoglobin antibody recognizes only plakoglobin 1a of the two paralogues and was validated by western blot [26]. Secondary antibody of goat-anti-mouse-biotin labeled IgG (1/300, Dako) was used. The signal was enhanced by the use of streptABComplex-horseradish peroxidase (Dako) and 3,3'-Diaminobenzidine (DAB) was used for visualization.

Data on tooth replacement were generated from the dissected branchial arches through immunohistochemistry on paraffin sections. After deparaffination and hydration, the sections were rinsed with PBS before blocking endogenous peroxidase in 1% H_2_O_2_ in MeOH for 45 minutes in the dark. Next, the sections were submerged in citrate buffer at 95°C for 20 minutes to retrieve the epitopes. After cooling to room temperature (RT), the sections were blocked with 1% Bovine serum albumin and 1% sheep serum in 1xPBS for 2h at RT and next incubated overnight at 4°C with the primary antibody. We used anti-plakoglobin clone 15 (1/500, BD Transduction Laboratories) or anti-β-catenin C7207 (1/300, Sigma). The latter was used by different authors (e.g. [59–61]) to immunolocalize beta-catenin without further distinction between the proteins of both paralogs. After washing, the secondary antibody goat-anti-mouse-biotin labelled IgG (1/300, Dako) or goat-anti-rabbit-biotin labelled IgG (1/300, Dako) was applied for 1h at RT. Afterwards, the sections were incubated with streptABComplex labelled with peroxidase (Dako) for 45 minutes at RT to increase the signal. Finally, 3,3'-Diaminobenzidine (DAB) was used for visualization.

## References

[1] Halbleib JM, Nelson WJ. Cadherins in development: cell adhesion, sorting, and tissue morphogenesis. Genes & Development. 2006;20(23):3199–214. . 17158740 10.1101/gad.1486806

[2] Munjal A, Lecuit T. Actomyosin networks and tissue morphogenesis. Development. 2014;141(9):1789–93. Epub 2014/04/24. 10.1242/dev.091645 24757001

[3] Niessen CM, Leckband D, Yap AS. Tissue organization by cadherin adhesion molecules: dynamic molecular and cellular mechanisms of morphogenetic regulation. Physiol Rev. 2011;91(2):691–731. Epub 2011/04/30. 10.1152/physrev.00004.2010 21527735 PMC3556819

[4] Takeichi M. Dynamic contacts: rearranging adherens junctions to drive epithelial remodelling. Nat Rev Mol Cell Biol. 2014;15(6):397–410. Epub 2014/05/16. 10.1038/nrm3802 24824068

[5] Ivanov DB, Philippova MP, Tkachuk VA. Structure and functions of classical cadherins. Biochemistry (Mosc). 2001;66(10):1174–86. . 11736639 10.1023/a:1012445316415

[6] Meng W, Takeichi M. Adherens junction: molecular architecture and regulation. Cold Spring Harb Perspect Biol. 2009;1(6):a002899 Epub 2010/05/12. 10.1101/cshperspect.a002899 20457565 PMC2882120

[7] van Roy F, Berx G. The cell-cell adhesion molecule E-cadherin. Cell Mol Life Sci. 2008;65(23):3756–88. 10.1007/s00018-008-8281-1 18726070 PMC11131785

[8] Garrod D, Chidgey M. Desmosome structure, composition and function. Biochim Biophys Acta. 2008;1778(3):572–87. Epub 2007/09/15. 10.1016/j.bbamem.2007.07.014 17854763

[9] Harmon RM, Green KJ. Structural and functional diversity of desmosomes. Cell Commun Adhes. 2013;20(6):171–87. Epub 2013/11/12. 10.3109/15419061.2013.855204 24205984

[10] Zhurinsky J, Shtutman M, Ben-Ze'ev A. Plakoglobin and beta-catenin: protein interactions, regulation and biological roles. Journal of Cell Science. 2000;113(18):3127–39. . 10954412 10.1242/jcs.113.18.3127

[11] Bierkamp C, Schwarz H, Huber O, Kemler R. Desmosomal localization of beta-catenin in the skin of plakoglobin null-mutant mice. Development. 1999;126(2):371–81. Epub 1998/12/16. 9847250 10.1242/dev.126.2.371

[12] Choi HJ, Gross JC, Pokutta S, Weis WI. Interactions of plakoglobin and beta-catenin with desmosomal cadherins: basis of selective exclusion of alpha- and beta-catenin from desmosomes. J Biol Chem. 2009;284(46):31776–88. Epub 2009/09/18. 10.1074/jbc.M109.047928 19759396 PMC2797248

[13] Behrens J. Cadherins and catenins: role in signal transduction and tumor progression. Cancer Metastasis Rev. 1999;18(1):15–30. . 10505543 10.1023/a:1006200102166

[14] Bienz M. beta-Catenin: a pivot between cell adhesion and Wnt signalling. Curr Biol. 2005;15(2):R64–7. Epub 2005/01/26. 10.1016/j.cub.2004.12.058 15668160

[15] Nelson WJ, Nusse R. Convergence of Wnt, beta-catenin, and cadherin pathways. Science. 2004;303(5663):1483–7. Epub 2004/03/06. 10.1126/science.109429 15001769 PMC3372896

[16] Haegel H, Larue L, Ohsugi M, Fedorov L, Herrenknecht K, Kemler R. Lack of beta-catenin affects mouse development at gastrulation. Development. 1995;121(11):3529–37. Epub 1995/11/01. 8582267 10.1242/dev.121.11.3529

[17] Ruiz P, Brinkmann V, Ledermann B, Behrend M, Grund C, Thalhammer C, et al Targeted mutation of plakoglobin in mice reveals essential functions of desmosomes in the embryonic heart. J Cell Biol. 1996;135(1):215–25. Epub 1996/10/01. 8858175 10.1083/jcb.135.1.215 PMC2121015

[18] Borday-Birraux V, Van der Heyden C, Debiais-Thibaud M, Verreijdt L, Stock DW, Huysseune A, et al Expression of Dlx genes during the development of the zebrafish pharyngeal dentition: evolutionary implications. Evol Dev. 2006;8(2):130–41. . 16509892 10.1111/j.1525-142X.2006.00084.x

[19] Lesot H, Brook AH. Epithelial histogenesis during tooth development. Arch Oral Biol. 2009;54 Suppl 1:S25–33. Epub 2008/07/29. 10.1016/j.archoralbio.2008.05.019 18656852

[20] Tucker AS, Fraser GJ. Evolution and developmental diversity of tooth regeneration. Semin Cell Dev Biol. 2014;25–26:71–80. Epub 2014/01/11. 10.1016/j.semcdb.2013.12.013 24406627

[21] Van der Heyden C, Huysseune A. Dynamics of tooth formation and replacement in the zebrafish (Danio rerio) (Teleostei, Cyprinidae). Dev Dyn. 2000;219(4):486–96. . 11084648 10.1002/1097-0177(2000)9999:9999<::AID-DVDY1069>3.0.CO;2-Z

[22] Verstraeten B, Sanders E, van Hengel J, Huysseune A. Zebrafish teeth as a model for repetitive epithelial morphogenesis: dynamics of E-cadherin expression. BMC Dev Biol. 2010;10:58 10.1186/1471-213X-10-58 20515472 PMC2890594

[23] Nanba D, Nakanishi Y, Hieda Y. Changes in adhesive properties of epithelial cells during early morphogenesis of the mammary gland. Dev Growth Differ. 2001;43(5):535–44. . 11576170 10.1046/j.1440-169x.2001.00596.x

[24] Kieffer-Combeau S, Meyer JM, Lesot H. Cell-matrix interactions and cell-cell junctions during epithelial histo-morphogenesis in the developing mouse incisor. Int J Dev Biol. 2001;45(5–6):733–42. . 11669375

[25] Obara N, Lesot H. Subcellular localization of beta-catenin and cadherin expression in the cap-stage enamel organ of the mouse molar. Histochem Cell Biol. 2004;121(4):351–8. . 14997319 10.1007/s00418-004-0637-5

[26] Martin ED, Moriarty MA, Byrnes L, Grealy M. Plakoglobin has both structural and signalling roles in zebrafish development. Developmental Biology. 2009;327(1):83–96. 10.1016/j.ydbio.2008.11.036 . 19101534

[27] Bellipanni G, Varga M, Maegawa S, Imai Y, Kelly C, Myers AP, et al Essential and opposing roles of zebrafish beta-catenins in the formation of dorsal axial structures and neurectoderm. Development. 2006;133(7):1299–309. Epub 2006/03/03. 10.1242/dev.02295 16510506

[28] Martin ED, Grealy M. Plakoglobin expression and localization in zebrafish embryo development. Biochemical Society Transactions. 2004;32:797–8. . 15494018 10.1042/BST0320797

[29] Falk MM. Adherens junctions remain dynamic. BMC Biol. 2010;8:34 Epub 2010/04/10. 10.1186/1741-7007-8-34 ; PubMed Central PMCID: PMC2867777. 20377885 PMC2867777

[30] Van der heyden C, Huysseune A, Sire JY. Development and fine structure of pharyngeal replacement teeth in juvenile zebrafish (Danio rerio) (Teleostei, Cyprinidae). Cell Tissue Res. 2000;302(2):205–19. . 11131132 10.1007/s004410000180

[31] Lo Muzio L, Lo Russo L, Pannone G, Santoro A, Leonardi R, Serpico R, et al Expression of beta-catenin in human tooth germ. Anal Quant Cytol Histol. 2009;31(5):324–31. Epub 2010/08/13. 20701100

[32] Obara N, Suzuki Y, Takeda M. Gene expression of beta-catenin is up-regulated in inner dental epithelium and enamel knots during molar tooth morphogenesis in the mouse. Cell Tissue Res. 2006;325(1):197–201. Epub 2006/03/22. 10.1007/s00441-005-0136-6 16550360

[33] Wang B, Li H, Liu Y, Lin X, Lin Y, Wang Y, et al Expression patterns of WNT/beta-CATENIN signaling molecules during human tooth development. J Mol Histol. 2014;45(5):487–96. Epub 2014/03/22. 10.1007/s10735-014-9572-5 24647585

[34] Verstraeten B, Sanders E, van Hengel J, Huysseune A. Expression pattern of E-cadherin during development of the first tooth in zebrafish (Danio rerio). Journal of Applied Ichthyology. 2010;26(2):202–4. 10.1111/j.1439-0426.2010.01405.x .

[35] Nanba D, Hieda Y, Nakanishi Y. Remodeling of desmosomal and hemidesmosomal adhesion systems during early morphogenesis of mouse pelage hair follicles. J Invest Dermatol. 2000;114(1):171–7. . 10620134 10.1046/j.1523-1747.2000.00842.x

[36] Da Silva L, Parry S, Reid L, Keith P, Waddell N, Kossai M, et al Aberrant expression of E-cadherin in lobular carcinomas of the breast. Am J Surg Pathol. 2008;32(5):773–83. 10.1097/PAS.0b013e318158d6c5 18379416

[37] Sundfeldt K. Cell-cell adhesion in the normal ovary and ovarian tumors of epithelial origin; an exception to the rule. Mol Cell Endocrinol. 2003;202(1–2):89–96. . 12770736 10.1016/s0303-7207(03)00068-6

[38] Huysseune A. Formation of a successional dental lamina in the zebrafish (Danio rerio): support for a local control of replacement tooth initiation. Int J Dev Biol. 2006;50(7):637–43. . 16892177 10.1387/ijdb.052098ah

[39] Laurenti P, Thaeron C, Allizard F, Huysseune A, Sire JY. Cellular expression of eve1 suggests its requirement for the differentiation of the ameloblasts and for the initiation and morphogenesis of the first tooth in the zebrafish (Danio rerio). Dev Dyn. 2004;230(4):727–33. . 15254906 10.1002/dvdy.20080

[40] Fukunaga Y, Liu H, Shimizu M, Komiya S, Kawasuji M, Nagafuchi A. Defining the roles of beta-catenin and plakoglobin in cell-cell adhesion: isolation of beta-catenin/plakoglobin-deficient F9 cells. Cell Struct Funct. 2005;30(2):25–34. Epub 2005/12/17. 16357441 10.1247/csf.30.25

[41] Teuliere J, Faraldo MM, Shtutman M, Birchmeier W, Huelsken J, Thiery JP, et al beta-catenin-dependent and -independent effects of DeltaN-plakoglobin on epidermal growth and differentiation. Mol Cell Biol. 2004;24(19):8649–61. Epub 2004/09/16. 10.1128/MCB.24.19.8649-8661.2004 15367683 PMC516731

[42] Wickline ED, Du Y, Stolz DB, Kahn M, Monga SP. gamma-Catenin at adherens junctions: mechanism and biologic implications in hepatocellular cancer after beta-catenin knockdown. Neoplasia. 2013;15(4):421–34. Epub 2013/04/05. 23555187 10.1593/neo.122098 PMC3612914

[43] Schnittler HJ, Puschel B, Drenckhahn D. Role of cadherins and plakoglobin in interendothelial adhesion under resting conditions and shear stress. Am J Physiol. 1997;273(5 Pt 2):H2396-405. Epub 1997/12/31. 10.1152/ajpheart.1997.273.5.H2396 9374777

[44] Logan CY, Nusse R. The Wnt signaling pathway in development and disease. Annu Rev Cell Dev Biol. 2004;20:781–810. Epub 2004/10/12. 10.1146/annurev.cellbio.20.010403.113126 . 15473860

[45] Chen J, Lan Y, Baek JA, Gao Y, Jiang R. Wnt/beta-catenin signaling plays an essential role in activation of odontogenic mesenchyme during early tooth development. Dev Biol. 2009;334(1):174–85. Epub 2009/07/28. 10.1016/j.ydbio.2009.07.015 19631205 PMC2752344

[46] Jarvinen E, Salazar-Ciudad I, Birchmeier W, Taketo MM, Jernvall J, Thesleff I. Continuous tooth generation in mouse is induced by activated epithelial Wnt/beta-catenin signaling. Proc Natl Acad Sci U S A. 2006;103(49):18627–32. . 17121988 10.1073/pnas.0607289103 PMC1693713

[47] Jernvall J, Thesleff I. Reiterative signaling and patterning during mammalian tooth morphogenesis. Mech Dev. 2000;92(1):19–29. . 10704885 10.1016/s0925-4773(99)00322-6

[48] Kratochwil K, Dull M, Farinas I, Galceran J, Grosschedl R. Lef1 expression is activated by BMP-4 and regulates inductive tissue interactions in tooth and hair development. Genes Dev. 1996;10(11):1382–94. Epub 1996/06/01. 8647435 10.1101/gad.10.11.1382

[49] Liu F, Chu EY, Watt B, Zhang Y, Gallant NM, Andl T, et al Wnt/beta-catenin signaling directs multiple stages of tooth morphogenesis. Dev Biol. 2008;313(1):210–24. Epub 2007/11/21. 10.1016/j.ydbio.2007.10.016 18022614 PMC2843623

[50] Liu F, Dangaria S, Andl T, Zhang Y, Wright AC, Damek-Poprawa M, et al beta-Catenin initiates tooth neogenesis in adult rodent incisors. J Dent Res. 2010;89(9):909–14. Epub 2010/06/10. 10.1177/0022034510370090 20530729 PMC3148824

[51] Liu F, Millar SE. Wnt/beta-catenin signaling in oral tissue development and disease. J Dent Res. 2010;89(4):318–30. Epub 2010/03/05. 10.1177/0022034510363373 20200414 PMC3140915

[52] Sarkar L, Sharpe PT. Expression of Wnt signalling pathway genes during tooth development. Mech Dev. 1999;85(1–2):197–200. Epub 1999/07/23. doi: S0925-4773(99)00095-7 [pii]. 10415363 10.1016/s0925-4773(99)00095-7

[53] Huysseune A, Soenens M, Elderweirdt F. Wnt signaling during tooth replacement in zebrafish (Danio rerio): pitfalls and perspectives. Front Physiol. 2014;5:386 Epub 2014/10/24. 10.3389/fphys.2014.00386 25339911 PMC4186270

[54] Charpentier E, Lavker RM, Acquista E, Cowin P. Plakoglobin suppresses epithelial proliferation and hair growth in vivo. Journal of Cell Biology. 2000;149(2):503–19. . 10769039 10.1083/jcb.149.2.503 PMC2175163

[55] Klymkowsky MW, Williams BO, Barish GD, Varmus HE, Vourgourakis YE. Membrane-anchored plakoglobins have multiple mechanisms of action in Wnt signaling. Mol Biol Cell. 1999;10(10):3151–69. Epub 1999/10/08. 10512857 10.1091/mbc.10.10.3151 PMC25571

[56] Westerfield M. The Zebrafish Book A Guide for the Laboratory Use of Zebrafish (*Danio rerio*). 3rd Edition ed: University of Oregon Press; 1995. 385 p.

[57] Strahle U, Scholz S, Geisler R, Greiner P, Hollert H, Rastegar S, et al Zebrafish embryos as an alternative to animal experiments—a commentary on the definition of the onset of protected life stages in animal welfare regulations. Reprod Toxicol. 2012;33(2):128–32. Epub 2011/07/06. 10.1016/j.reprotox.2011.06.121 21726626

[58] Verstraeten B, Sanders E, Huysseune A. Whole mount immunohistochemistry and *in situ* hybridization of larval and adult zebrafish dental tissues**.** In: Kioussi C, editor. Methods in Molecular Biology, Methods in Odontogenesis: Humana Press, USA; 2012 p. 179–91. 10.1007/978-1-61779-860-3_16 22566056

[59] Garavito-Aguilar ZV, Riley HE, Yelon D. Hand2 ensures an appropriate environment for cardiac fusion by limiting Fibronectin function. Development. 2010;137(19):3215–20. Epub 2010/08/21. 10.1242/dev.052225 20724450 PMC2934734

[60] Song S, Eckerle S, Onichtchouk D, Marrs JA, Nitschke R, Driever W. Pou5f1-dependent EGF expression controls E-cadherin endocytosis, cell adhesion, and zebrafish epiboly movements. Dev Cell. 2013;24(5):486–501. Epub 2013/03/15. 10.1016/j.devcel.2013.01.016 23484854 PMC3598594

[61] Yang J, Xu X. Immunostaining of dissected zebrafish embryonic heart. J Vis Exp. 2012;(59):e3510 Epub 2012/01/20. 10.3791/3510 22258109 PMC3369776

